# Prognostic significance of multiparametric flow cytometry minimal residual disease at two time points after induction in pediatric acute myeloid leukemia

**DOI:** 10.1186/s12885-023-11784-4

**Published:** 2024-01-09

**Authors:** Yongzhi Zheng, Lili Pan, Jian Li, Xiaoqin Feng, Chunfu Li, Mincui Zheng, Huirong Mai, Lihua Yang, Yingyi He, Xiangling He, Honggui Xu, Hong Wen, Shaohua Le

**Affiliations:** 1 Department of Pediatric Hematology, Fujian Institute of Hematology, Fujian Provincial Key Laboratory On Hematology, Fujian Medical University Union Hospital, Fuzhou, China; 2 Department of Pediatrics, Nanfang Hospital, Southern Medical University, Guangzhou, China; 3 Nanfang-Chunfu Children’s Institute of Hematology & Oncology, TaiXin Hospital, Dongguan, China; 4 Department of Pediatric Hematology/Oncology, Hematology and Oncology, Hunan Children’s Hospital, Changsha, China; 5 Department of Pediatric Hematology/Oncology, Shenzhen Children’s Hospital, Shenzhen, China; 6 Department of Pediatrics, Zhujiang Hospital of Southern Medical University, Guangzhou, China; 7 Department of Pediatric Hematology/Oncology, Guangzhou Women and Children’s Medical Center, Guangzhou, China; 8 People’s Hospital of Hunan Province, Changsha, China; 9 Sun Yat-Sen Memorial Hospital, Guangzhou, China; 10 The First Affiliated Hospital of Xiamen University, Xiamen, China

**Keywords:** Pediatric acute myeloid leukemia, Minimal residual disease, Multiparametric flow cytometry, Prognostic value

## Abstract

**Background:**

Prompt response to induction chemotherapy is a prognostic factor in pediatric acute myeloid leukemia. In this study, we aimed to evaluate the prognostic significance of multiparametric flow cytometry-minimal residual disease (MFC-MRD), assessed at the end of the first and second induction courses.

**Methods:**

MFC-MRD was performed at the end of the first induction (TP1) in 524 patients and second induction (TP2) in 467 patients who were treated according to the modified Medical Research Council (UK) acute myeloid leukemia 15 protocol.

**Results:**

Using a 0.1% cutoff level, patients with MFC-MRD at the two time points had lower event-free survival and overall survival. Only the TP2 MFC-MRD level could predict the outcome in a separate analysis of high and intermediate risks based on European LeukemiaNet risk stratification and *KMT2A* rearrangement. The TP2 MFC-MRD level could further differentiate the prognosis of patients into complete remission or non-complete remission based on morphological evaluation. Multivariate analysis indicated the TP2 MFC-MRD level as an independent adverse prognostic factor for event-free survival and overall survival. When comparing patients with MFC-MRD ≥ 0.1%, those who underwent hematopoietic stem cell transplant during the first complete remission had significantly higher 5-year event-free survival and overall survival and lower cumulative incidence of relapse than those who only received consolidation chemotherapy.

**Conclusions:**

The TP2 MFC-MRD level can predict the outcomes in pediatric patients with acute myeloid leukemia and help stratify post-remission treatment.

**Supplementary Information:**

The online version contains supplementary material available at 10.1186/s12885-023-11784-4.

## Background

The current survival rates for children with acute myeloid leukemia (AML) treated in clinical trials conducted in high-income countries have improved by over 75%, mostly owing to improvements in supportive care and risk stratification of therapy [1]. Genomic complexity is considered the underlying reason for the suboptimal outcome in AML, and molecular characteristics such as cytogenetics and mutations are the main basis for risk stratification [2, 3]. However, the prognosis of patients with the same genetic abnormality but different early treatment responses may still show substantial variations [4, 5]. Furthermore, a subset of patients lacks risk-associated molecular markers. Consequently, early response to therapy has emerged as an increasingly essential tool for risk stratification and guiding post-remission therapeutic strategies [6, 7].

Although the morphological assessment of early response after the first induction treatment, i.e., the presence of < 5% residual leukemia blasts, is a strong predictor for treatment outcome, it has low sensitivity and poor specificity for accurate determination of the disease status [8, 9]. In AML, the assessment of minimal residual disease (MRD), which allows the identification of 0.1%–0.001% leukemic cells, can establish a more detailed remission status than morphology-based evaluation, and it improves outcome prediction [10]. Different detection techniques are currently available for MRD in pediatric AML, including quantitative analysis of specific gene fusions using RNA-based reverse transcription polymerase chain reaction (RT-PCR) and multiparametric flow cytometry (MFC) for detecting aberrant immunophenotypes [11, 12]. RT-PCR of fusion transcripts allows MRD assessment with a sensitivity of 0.01%–0.001%, although it is applicable only in 50%–60% cases with pediatric AML with a detectable fusion gene or mutations [12]. Although MFC-MRD has lower sensitivity than RT-PCR (up to 0.1%–0.01%), it is the only method that can be used in almost all patients with childhood AML [7]. Therefore, MFC-MRD is generally the preferred method for MRD detection in clinical AML studies.

To date, several reports have demonstrated that the detection of MFC-MRD during treatment can predict the final outcomes of patients [8, 13–15]. However, only one study [7] documented that MFC-MRD evaluation can be instrumental for the prospective stratification of pediatric patients with AML and guide post-remission therapy. There is a need for a multicenter study on the prospective use of MFC-MRD to stratify patients into different classes of risk. The clinical application of MFC-MRD in pediatric AML started relatively late in China; therefore, a multicenter cohort study of Chinese children is lacking. Here, we retrospectively analyzed the data of a large group of children with de novo AML, treated following the modified Medical Research Council (UK) AML 15 protocol (named C-HUANAN-AML 15 study). We also detected MFC-MRD in a centralized laboratory in China to evaluate the prognostic significance of MFC-MRD, assessed at the end of the first and second induction courses.

## Methods

### Patients

From January 2015 to December 2020, 584 patients aged < 14 years who were newly diagnosed with AML, were enrolled in the C-HUANAN-AML 15 study at 10 centers in southern China. The 10 centers in 7 cities were hematology departments of children’s hospitals or hematology divisions of pediatric departments in general university hospitals. The number of patients enrolled at each center is shown in Supplementary Table 1, Additional file. Morphological, flow cytometric, cytogenetic, and molecular analyses were performed on all patients upon diagnosis, and the results were available for all patients included in this study. AML was diagnosed based on the morphological assessments of the bone marrow, outlined in the French-American-British and World Health Organization classifications [16]. The characteristics of the patients enrolled are summarized in Supplementary Table 2, Additional file.

Informed consent was obtained from parents or legal guardians according to the Declaration of Helsinki, and the treatment protocol was approved by the Ethic Committee of Fujian Medical University Union Hospital.

### C-HUANAN-AML 15 protocol

The protocol was designed based on the Medical Research Council AML 15 trial with some adjustments and named the C-HUANAN-AML 15 protocol. Chemotherapy included only four courses: two tandem courses of the FLAG-IDA or DAE regimen as induction chemotherapy, one course of homoharringtonine cytarabine/etoposide (amsacrine used in the Medical Research Council AML 15 trial was replaced by homoharringtonine in the protocol as amsacrine is not sold in China), and one course of mitoxantrone/cytarabine as consolidation. The division of patients into A group (FLAG-IDA induction) or B group (DAE induction) was non-random. The details of treatment protocols are shown in Supplementary Fig. 1, Additional file.

Central nervous system (CNS)-directed therapy was achieved using four courses of “triple” intrathecal therapy (methotrexate, cytarabine, and hydrocortisone) in age-adjusted doses, one after each chemotherapy course. Children with CNS disease at diagnosis received six additional triple intrathecal treatments each week until the cerebrospinal fluid was clear. Children aged ≥ 2 years with CNS disease who did not undergo hematopoietic stem cell transplantation were recommended to receive cranial irradiation (CRT, 18 Gy total, divided 10–15 times in 2–3 weeks) after the final course of chemotherapy, except those receiving total body irradiation as part of hematopoietic stem cell transplantation (HSCT) conditioning. Children aged < 2 years were not eligible for CRT.

Risk group stratification based on genetic abnormalities and findings of morphological assessment of early response after induction treatment are shown in Supplementary Table 3, Additional file. Patients with *AML1-ETO*, *CBFB-MYH11*, *NPM1*, or isolated biallelic (double) *CEBPA* mutation in the absence of *FLT3-ITD*, who achieved CR after the first induction course were stratified as the low-risk (LR) group. Patients with mutated *FLT3-ITD*, complex karyotype, -5 or del(5q), abn(3q), abn(17p), -7 or del(7q), and ≥ 15% blast in bone marrow at the end of the first induction course or no complete remission (CR) after the second induction course irrespective of genetic abnormalities were stratified as the high-risk (HR) group. After excluding LR or HR patients with genetic abnormality and those with blast in bone at a rate of < 15% after the first induction course and CR after the second induction irrespective of genetic abnormalities, patients were stratified as the intermediate-risk (IR) group. LR and IR patients without a sibling donor were advised to receive chemotherapy only. In contrast, IR patients with a sibling donor and all HR patients were advised to undergo HSCT.

### Multiparametric flow cytometric evaluation of MRD

**Fig. 1:**
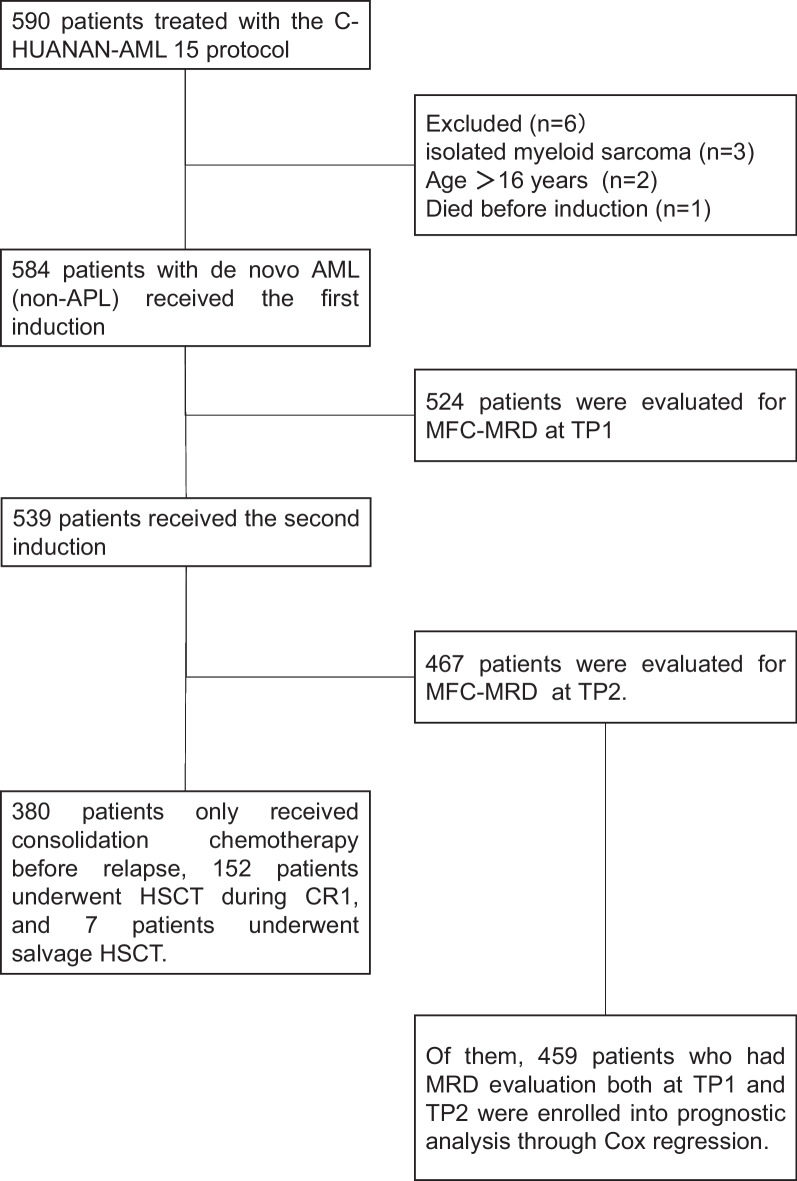
Outline of patient enrollment in this study. AML, acute myeloid leukemia; APL, acute promyelocytic leukemia; TP1, on days 28–35 after the first induction course; TP2, at the end of the second induction course (before start of consolidation); MFC, multiparametric flow cytometry; MRD, minimal residual disease; HSCT, hematopoietic stem cell transplantation; CR1, first complete remission

At the time of diagnosis, bone marrow samples from all patients were assessed using 8-color MFC assays containing antibodies against the markers enumerated in Supplementary Table 4, Additional file. During days 28–35 following the first induction course (referred to as time point 1 [TP1]), bone marrow samples were collected from a total of 524 patients to evaluate MFC-MRD. Similarly, after the second induction course, just before the consolidation therapy (referred to as time point 2 [TP2]), bone marrow samples were obtained from 467 patients for the assessment of MFC-MRD (see Fig. 1). MFC-MRD analyses conducted on patients from various hospitals (the detailed hospital name is listed in Supplementary Table 5) were conducted at a centralized laboratory in China, specifically at Kindstar Globalgene Technology, Inc, in Beijing. The FACSCanto instruments (Beckton Dickinson, Franklin Lakes, NJ, USA) were utilized for the analyses. The MFC-MRD analyses performed on patients from other hospitals (the detailed hospital name is listed in the Supplementary Table 5) were carried out at a centralized laboratory in China, specifically at KingMed Diagnostics Group Co., Ltd. in Guangzhou. The NAVIOS instruments (Beckman Coulter, Bria, CA, USA) were utilized for this purpose.

Specimens were processed with the same procedure used at diagnosis but 10^6^ cells were used for each tube. MRD was assessed through 5-color MFC, acquiring at least 500 000 events for each tube [17].

Two complementary approaches were employed to enhance the efficiency of identifying residual disease in monitoring leukemic populations. The first approach involved assessing the expression of specific leukemia-associated immunophenotypes (LAIPs) during diagnosis, followed by continuous monitoring of these original LAIPs during post-therapy follow-up. The second approach, known as the different-from-normal approach, focused on the abnormal differentiation and maturation patterns observed during follow-up [10, 17, 18].

The monoclonal antibodies often employed in five-color combinations for the detection of MFC-MRD are included in Supplementary Table 6, Additional file. Nevertheless, owing to the varied nature of AML, the selection of MFC-MRD detection antibodies is often personalized. To mitigate the risk of phenotypic shifts leading to false negatives, we employed a strategy wherein each patient was subjected to a range of 3–5 antigen combinations. A cluster of at least 50 events (among the 500,000 acquired events) was considered to distinguish between leukemia cells and normal cells.

### Statistical analyses

Overall survival (OS) was calculated from the date of diagnosis to the time of death due to any cause or the time of the last contact.

Event-free survival (EFS) was calculated from the date of diagnosis to the last follow-up or first event (failure to achieve CR or CRi after second induction, relapse, secondary malignancy, or death due to any cause, whichever occurred first).

CR was defined as bone marrow with < 5% leukemic cells and evidence of regeneration of normal hemopoietic cells; CRi was defined as bone marrow with < 5% leukemic cells, although neutrophil and platelet parameters were not incomplete recoveries [19].

The probabilities of OS and EFS were estimated using the Kaplan–Meier method. Differences between groups were evaluated using the log-rank test. The cumulative incidence of relapse (CIR) was estimated, considering death in remission as the competing event. The Gray test was performed to assess differences between cumulative incidence in univariate analyses.

Continuous variables of patient characteristics were compared using the Wilcoxon rank sum test (non-normal distribution) or Mann–Whitney test (normal distribution), whereas categorical variables were compared using the Pearson chi-squared test or Fisher exact test when data were sparse. Primary analyses were conducted using intention-to-treat analysis. Univariate analyses were performed using the unadjusted Cox proportional hazards model to calculate hazard ratios (HRs). Variables that were significant in univariate analyses were included in multivariate analyses. Multivariate analyses were performed using the Cox proportional hazards model to identify independent prognostic factors. All tests were two-sided, and a *P*-value of < 0.05 was considered statistically significant. Statistical analyses were performed using SPSS v25.0 (SPSS Inc., Chicago, IL, USA). Graphs were constructed using GraphPad Prism version 7 (GraphPad Software, San Diego, CA, USA).

## Results

### MFC-MRD characteristics at TP1 and TP2

**Fig. 2:**
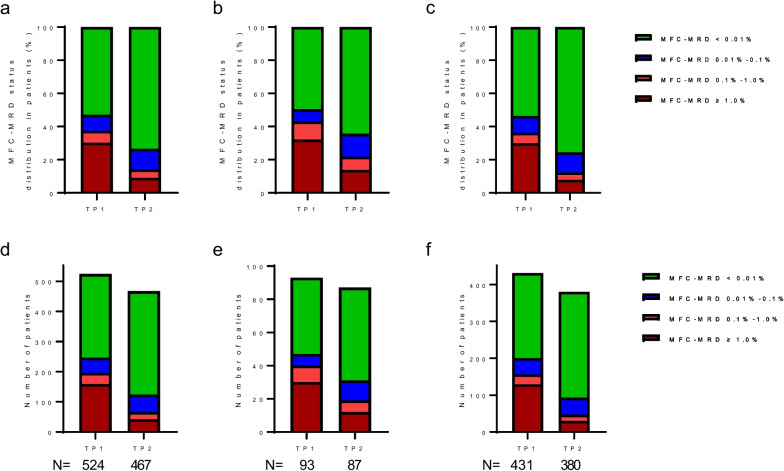
MFC-MRD levels at TP1 and TP2.MFC-MRD levels at TP1 and TP2 in the whole cohort (**a**, **d**), in relapsed patients (**b**, **e**), and in patients who had not relapsed (**c**, **f**). MFC, multiparametric flow cytometry; MRD, minimal residual disease; TP1, on days 28–35 after the first induction course; TP2, at the end of the second induction course (before start of consolidation)

The levels of MFC-MRD detected at TP1 were assessed in a total of 524 individuals who had samples available for MFC-MRD analysis. Among these patients, 277 had MFC-MRD levels below 0.01%, 51 had levels between 0.01% and 0.1%, 37 had levels between 0.1% and 1%, and 159 had levels equal to or greater than 1%. Additionally, MFC-MRD evaluation was performed in a total of 467 patients. The number of patients with MRD < 0.01%, ≥ 0.01% to < 0.1%, ≥ 0.1% to < 1%, and ≥ 1% was 343, 58, 24 and 42, respectively. The levels of MFC-MRD at TP1 and TP2 in both relapsed and non-relapsed patients throughout the whole cohort are shown in Fig. 2a–f. With a threshold value of 0.1%, the frequency of MFC-MRD positive at TP1 and TP2 was higher in relapsed patients than in non-relapsed patients (TP1: 43.0% *vs*. 36.2% *P* < 0.001; TP2: 21.8% *vs*. 12.4%, *P* = 0.022).

At TP1 and TP2, 37.4% and 14.1% of patients, respectively, with an informative immunophenotype, tested positive for MFC-MRD using a threshold level of 0.1%. Patients with positive and negative MFC-MRD showed similar clinical characteristics at diagnosis, except a higher proportion of white blood cells (≥ 50 × 10^9^/L) and CNSL at diagnosis and HR factors (according to the C-HUANAN-AML 15 criteria) in patients with positive MFC-MRD and the presence of *RUNX1-RUNX1T1* fusion gene in patients with negative MFC-MRD. In addition, a substantially higher proportion of patients with a positive MFC-MRD at TP2 received HSCT as their primary therapy (Table 1).

**Table 1:** Characteristics of the patients according to minimal residual disease (MRD) in TP1 and TP2

Characteristics	MRD after the first induction course (*n* = 524)				MRD before start of consolidation (*n* = 467)			
	All	< 0.1%	≥ 0.1%	*P*	All	< 0.1%	≥ 0.1%	*P*
Sex, n (%)				0.453				0.164
Male	313 (59.7)	200 (61.0)	113 (57.7)		277 (59.3)	243 (60.6)	34 (51.5)	
Female	211 (40.3)	128 (39.0)	83 (42.3)		190 (40.7)	158 (39.4)	32 (48.5)	
Age, n (%)				0.677				0.075
< 10 years	412 (78.6)	256 (78.0)	156 (79.6)		364 (77.9)	307 (76.6)	57 (86.4)	
≥ 10 years	112 (21.4)	72 (22.0)	40 (20.4)		103 (22.1)	94 (23.4)	9 (13.6)	
WBC at diagnosis, n (%)				0.175				0.028
≥ 50 × 10^9^/L	158 (30.2)	92 (28.0)	66 (33.7)		144 (30.8)	116 (28.9)	28 (42.4)	
< 50 × 10^9^/L	366 (69.8)	236 (72.0)	130 (66.3)		323 (69.2)	285 (71.1)	38 (57.6)	
CNSL at diagnosis, n (%)	18 (3.6)	9 (2.7)	9 (4.6)	0.261	17 (3.6)	12 (3.0)	5 (7.6)	0.020
FAB classification, n (%)				0.910				0.541
M7	41 (7.8)	26 (7.9)	15 (7.7)		34 (7.3)	28 (7.0)	6 (9.1)	
Non-M7	483 (92.2)	302 (92.1)	181 (92.3)		433 (92.7)	373 (93.0)	60 (90.9)	
Genetic subgroups, n (%)								
*RUNX1-RUNX1T1*	139 (26.5)	94 (28.7)	45 (23.0)	0.153	127 (27.2)	114 (28.4)	9 (13.6)	0.011
*CBFB-MYH11*	35 (6.7)	24 (7.3)	11 (5.6)	0.439	33 (7.1)	29 (7.2)	4 (6.1)	0.929
*KMT2A-*r excluded *MLLT3-KMT2A*	56 (10.7)	33 (10.1)	23 (11.7)	0.548	49 (10.5)	40 (10.0)	9 (13.6)	0.368
*MLLT3-KMT2A*	42 (8.0)	26 (7.9)	16 (8.2)	0.923	36 (7.7)	32 (8.0)	4 (6.1)	0.919
*FLT3-ITD* mutation	51 (9.7)	28 (8.5)	23 (11.7)	0.232	43 (9.2)	36 (9.0)	7 (10.6)	0.324
Risk stratification according to C-HUANAN-AML 15 criteria, n (%)				0.000				0.000
HR	114 (21.8)	52 (15.9)	62 (31.6)		96 (20.6)	67 (16.7)	29 (43.9)	
Non-HR	410 (78.2)	276 (84.1)	134 (68.4)		371 (79.4)	334 (83.3)	37 (56.1)	
Risk stratification according to 2017 ELN criteria, n (%)				0.156				0.135
HR	191 (36.3)	112 (34.1)	79 (40.3)		167 (35.8)	138 (34.4)	29 (43.9)	
Non-HR	333 (63.7)	216 (65.9)	117 (59.7)		300 (64.2)	263 (65.6)	37 (56.1)	
HSCT in primary therapy				0.057				0.009
Yes	156 (29.8)	88 (26.8)	68 (34.7)		135 (28.9)	107 (26.7)	28 (42.4)	
No	368 (70.2)	240 (73.2)	128 (65.3)		332 (71.1)	294 (73.3)	38 (57.6)	

*TP1* on days 28–35 after the first induction course, *TP2* at the end of the second induction course (before start of consolidation), *N* number, *WBC* white blood cell count, *FAB* French-American-British, *FLT3-ITD* FLT3 internal tandem duplication, *CNSL* central nervous system leukemia, *HR* high risk, *ELN* European LeukemiaNet, *HSCT* hematopoietic stem cell transplantation

### Prognostic significance of MFC-MRD at TP1 and TP2

The correlation between survival probability and MFC-MRD levels was evaluated in 524 patients with MFC-MRD data at TP1 and 467 patients with MFC-MRD data at TP2.

**Fig. 3:**
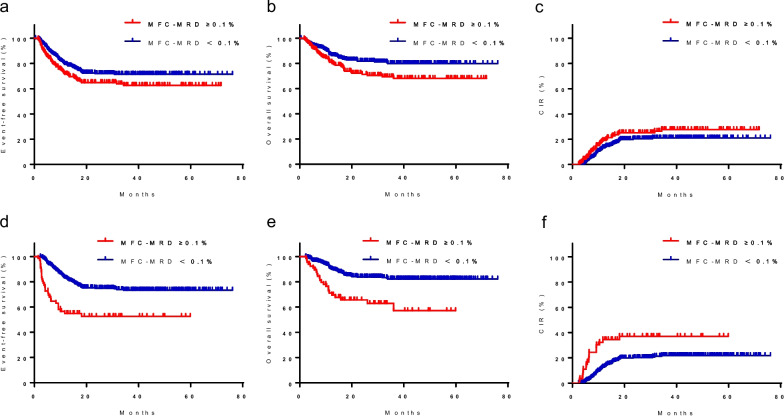
Survival probability by MFC-MRD status at TP1 and TP2. According to the MFC-MRD levels at TP1 and TP2, patients were stratified into two groups based on MFC-MRD (MFC-MRD < 0.1%; MFC-MRD ≥ 0.1%). EFS (**a**), OS (**b**), and CIR (**c**) according to MFC-MRD at TP1. EFS (**d**), OS (**e**), and CIR (**f**) according to MFC-MRD at TP2. EFS, event-free survival; OS, overall survival; CIR, cumulative incidence of relapse; MFC, multiparametric flow cytometry; MRD, minimal residual disease, TP1, on days 28–35 after the first induction course; TP2, at the end of the second induction course (before start of consolidation)

First, we stratified the patients into three groups according to MFC-MRD levels at TP1 (< 0.01%, 0.01%–0.1%, and ≥ 0.1%). The 5-year EFS of the three groups was not significantly different: 70.6% ± 3.0%, 75.1% ± 6.3%, and 63.8% ± 3.7%, respectively, (*P* = 0.098) (Supplementary Fig. 2a, Additional file). As patients in the MFC-MRD < 0.01% and MFC-MRD 0.01%–0.1% subgroups at TP1 had similar EFS, we considered grouping these patients together. The MFC-MRD ≥ 0.1% subgroup had a lower 5-year EFS and OS than the MFC-MRD < 0.1% subgroup (EFS: 63.8% ± 3.8% *vs*. 71.4% ± 2.7%, *P* = 0.034; OS: 68.0% ± 3.9% *vs*. 79.9% ± 2.5%, *P* = 0.009) (Fig. 3a, b). However, the 5-year CIR in the MFC-MRD ≥ 0.1% and MFC-MRD < 0.1% subgroups at TP1 was not significantly different: 27.7% ± 3.9% *vs*. 21.0% ± 2.6% (*P* = 0.112) (Fig. 3c).

Similarly, according to the MFC-MRD levels at TP2, patients were stratified into three groups (< 0.01%, 0.01%–0.1%, and ≥ 0.1%) with significantly different 5-year EFS, namely, 74.1% ± 2.7%, 68.7% ± 6.3%, and 52.5% ± 6.4%, respectively (*P* < 0.001) (Supplementary Fig. 2b, Additional file). As the 5-year EFS in the MFC-MRD < 0.01% subgroup was not significantly different from that in the MFC-MRD 0.01%–0.1% subgroup (74.8% ± 2.7% *vs*. 68.7% ± 6.3%, *P* = 0.283), we combined the results into a single group based on MFC-MRD levels. The MFC-MRD ≥ 0.1% subgroup had a lower 5-year EFS and OS and higher CIR (EFS: 52.5% ± 6.4% *vs*. 73.9% ± 2.5%, *P* < 0.001; OS: 57.1% ± 8.0% *vs*. 82.3% ± 2.2%, *P* = 0.112; CIR: 37.1% ± 6.9% *vs*. 21.80% ± 2.4%, *P* < 0.001) (Fig. 3d–f).

### Comparative prognostic values between MFC-MRD and morphological assessment

Among 453 patients with < 5% leukemic myeloblasts (< 5% blasts) detected based on morphology at TP1, 138 (30.5%) were MFC-MRD positive (≥ 0.1% by MFC), whereas 13 (18.3%) of the 71 patients with ≥ 5% blasts were MFC-MRD negative (< 0.1%). Among 431 patients with < 5% blasts detected based on morphology at TP2, 44 (10.2%) were MFC-MRD positive, whereas 14 (38.9%) of the 36 patients with ≥ 5% blasts were MFC-MRD negative.

Patients with < 5% blasts based on morphology at TP1 had a significantly higher 5-year EFS and OS and lower 5-year CIR than those with ≥ 5% blasts based on morphology (EFS: 72.0% ± 2.3% *vs*. 43.9% ± 6.2%, *P* < 0.001; OS: 78.8% ± 2.2% *vs*. 54.0% ± 7.0%, *P* < 0.001; CIR: 22.1% ± 2.3% *vs*. 35.3% ± 7.2%, *P* = 0.005) (Supplementary Fig. 3a–c, Additional file). Similarly, patients with ≥ 5% blasts based on morphology had a significantly higher 5-year EFS and OS and lower 5-year CIR than those with < 5% blasts based on morphology at TP2 (EFS: 73.5% ± 2.4% *vs*. 33.1% ± 7.9%, *P* < 0.001; OS: 81.6% ± 2.2% *vs*. 48.0% ± 9.3%, *P* < 0.001; CIR: 22.6% ± 2.3% *vs*. 45.8% ± 10.7%, *P* < 0.001) (Supplementary Fig. 3d–f, Additional file).

**Fig. 4:**
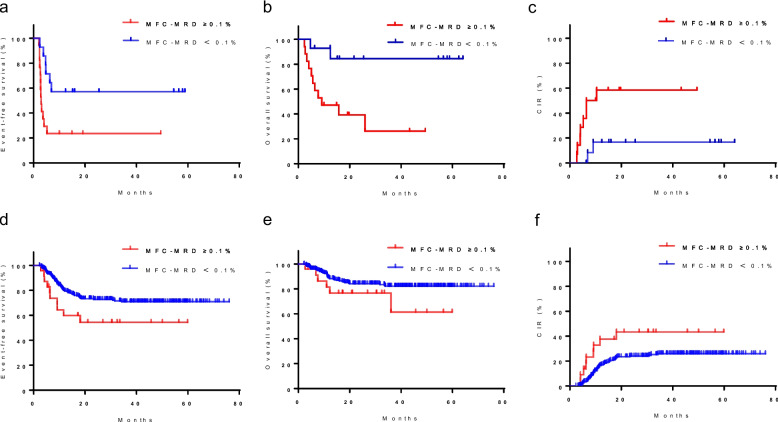
Survival probability by MFC-MRD status in patients with ≥ 5%/ < 5% blasts based on morphology at TP2. According to MFC-MRD levels at TP2, patients were stratified into two MFC-MRD-based groups (MFC-MRD < 0.1%; MFC-MRD ≥ 0.1%). EFS (**a**), OS (**b**), and CIR (**c**) of patients with ≥ 5% blasts by morphology according to MFC-MRD prior to the start of consolidation. EFS (**d**), OS (**e**), and CIR (**f**) of patients with < 5% blasts according to the MFC-MRD before consolidation. EFS, event-free survival; OS, overall survival; CIR, cumulative incidence of relapse; MFC, multiparametric flow cytometry; MRD, minimal residual disease, TP2, at the end of the second course of induction (before start of consolidation)

We then compared prognosis between the MFC-MRD ≥ 0.1% and MFC-MRD < 0.1% subgroup in a separate analysis of patients with < 5% or ≥ 5% blasts based on morphology at TP1 and TP2. Among patients with < 5% or ≥ 5% blasts based on morphology at TP1, the MFC-MRD ≥ 0.1% subgroup had 5-year EFS, OS, and CIR similar to those of the MFC-MRD < 0.1% subgroup (Supplementary Fig. 4a–f, Additional file). However, among patients with ≥ 5% blasts based on morphology at TP2, the MFC-MRD ≥ 0.1% subgroup had a lower 5-year EFS and OS and higher 5-year CIR than the MFC-MRD < 0.1% subgroup (EFS: 23.5% ± 10.3% *vs*. 57.1% ± 13.2%, *P* = 0.013; OS: 26.1% ± 13.5% *vs*. 84.4% ± 10.2%, *P* = 0.006; CIR: 58.3% ± 13.5% *vs*. 16.7% ± 10.8%, *P* = 0.020) (Fig. 4a–c). Similarly, in patients with < 5% blasts based on morphology at TP2, the MFC-MRD ≥ 0.1% subgroup had a lower 5-year EFS, similar to 5-year OS, and higher 5-year CIR than the MFC-MRD < 0.1% subgroup (EFS: 54.3% ± 10.8% *vs.* 70.9% ± 3.0%, *P* = 0.013; OS: 61.3% ± 15.6% *vs.* 82.2% ± 2.5%, *P* = 0.141; CIR: 43.3% ± 11.0% *vs.* 25.9% ± 3.0%, *P* = 0.028) (Fig. 4d–f).

### MFC-MRD at TP2 was an independent prognostic factor

We first performed a Cox regression univariate analysis in 459 patients with available MFC-MRD data at both TP1 and TP2, including sex, leucocyte count, *RUNX1-RUNX1T1*, *CBFB-MYH11*, *MLLT3-KMT2A*, *KMT2A* rearrangement excluding *MLLT3-KMT2A*, -7/7q-, *FLT3-ITD* mutations, *ASXL1* mutations, *NPM1* mutations, biallelic mutated *CEBPA*, and MFC-MRD ≥ 0.1% at TP2 (Table 2). Poor prognostic predictors included white blood cell counts ≥ 50 × 10^9^/L, the presence of *FLT3-ITD* mutation, and MFC-MRD levels ≥ 0.1% at TP1 and TP2. Conversely, *RUNX1-RUNX1T1* and *CBFB-MYH11*, considered as favorable factors, could predict the final outcomes.

**Table 2:** Univariate analysis by Cox regression of 459 patients with available MFC-MRD data at TP2

Risk factor	Overall survival			Event-free survival		
	HR	95% CI	*P-*value	HR	95% CI	*P-*value
Sex (male)	1.098	0.705–1.711	0.679	1.086	0.752–1.569	0.660
WBC at diagnosis (≥ 50 × 10^9^/L)	1.560	0.997–2.441	0.051	1.630	1.131–2.350	0.009
FAB classification (M7)	1.774	0.887–3.549	0.105	1.633	0.899–2.966	0.107
*RUNX1-RUNX1T1*	0.309	0.155–0.619	0.001	0.306	0.175–0.534	0.000
*CBFB-MYH11*	0.294	0.072–1.199	0.088	0.291	0.092–0.914	0.035
*MLLT3-KMT2A*	0.571	0.209–1.561	0.275	0.667	0.311–1.430	0.298
*KMT2A* rearrangement excluding *MLLT3-KMT2A*	1.599	0.866–2.955	0.134	1.292	0.752–2.220	0.353
-7 or 7q-	1.895	0.764–4.697	0.168	1.114	0.455–2.728	0.813
*FLT3-ITD* mutations	1.953	1.078–3.538	0.027	1.969	1.206–3.215	0.007
*ASXL1* mutations	0.770	0.282–2.104	0.610	0.908	0.423–1.948	0.804
*NPM1* mutations	1.401	0.344–5.705	0.638	0.943	0.233–3.817	0.935
Biallelic mutated *CEBPA*	0.340	0.047–2.447	0.284	0.680	0.216–2.140	0.510
MRD ≥ 0.1% before consolidation	2.882	1.774–4.683	0.000	2.703	1.787–4.088	0.000

*TP2* at the end of the second induction course (before start of consolidation), *WBC* white blood cell count, *FAB* French-American-British; MFC, multiparametric flow cytometry, *MRD* minimal residual disease, *HR* hazard ratio, *CI* confidence interval

Thereafter, we performed multivariate analysis by Cox regression that included factors associated with the outcome in univariate analysis (Table 3). MFC-MRD levels ≥ 0.1% at TP2, was an independent prognostic factor that affected OS [HR 2.190 (95% confidence interval [*CI*] 1.262–3.799), *P* = 0.005] and EFS [HR 2.311 (95% *CI* 1.441–3.706), *P* = 0.001]. In contrast, the presence of *RUNX1-RUNX1T1* was a favorable independent prognostic predictor for OS [HR 0.326 (95% *CI* 0.161–0.660), *P* = 0.002] and EFS [HR 0.314 (95% *CI* 0.178–0.553), *P* < 0.001].

**Table 3:** Multivariate analysis by Cox regression of 459 patients with available MFC-MRD data at both TP1 and TP2

Risk factor	Overall survival			Event-free survival		
	HR	95% *CI*	*P-*value	HR	95% *CI*	*P-*value
WBC at diagnosis (≥ 50 × 10^9^/L)	1.202	0.750–1.926	0.445	1.263	0.859–1.858	0.235
*RUNX1-RUNX1T1*	0.326	0.161–0.660	0.002	0.314	0.178–0.553	0.000
*CBFB-MYH11*	0.254	0.062–1.046	0.058	0.232	0.073–0.738	0.013
*FLT3-ITD* mutations	1.535	0.831–2.837	0.171	1.482	0.892–2.462	0.129
MRD ≥ 0.1% before start of consolidation	2.466	1.511–4.027	0.000	2.358	1.553–3.580	0.000

*TP2* at the end of the second induction course (before start of consolidation), *WBC* white blood cell count, *FAB* French-American-British, *MFC* multiparametric flow cytometry, *MRD* minimal residual disease, *HR* hazard ratio, *CI* confidence interval

### Prognostic significance of MFC-MRD in different risk stratification and some genetic subtypes

**Fig. 5:**
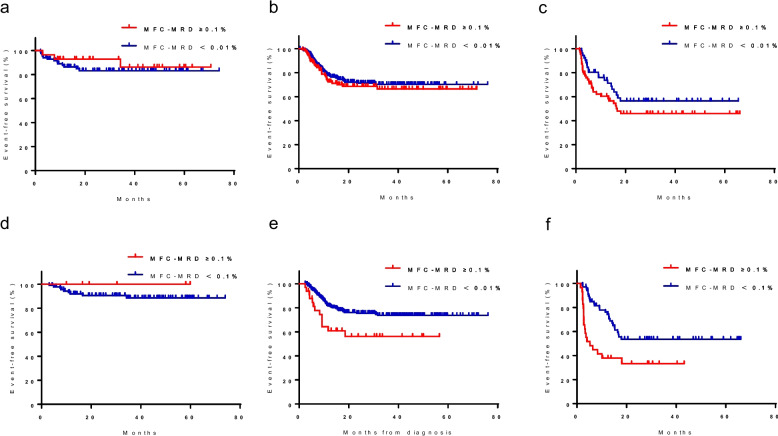
EFS by MFC-MRD status at TP1 and TP2, stratifying risks per the C-HUANAN-AML 15 protocol.According to MFC-MRD levels, patients were stratified into two MFC-MRD-based groups (MRD < 0.1%; MRD ≥ 0.1%). EFS according to MFC-MRD at TP1 in a separate analysis of LR (**a**), IR (**b**) and HR (**c**) patients. EFS according to MFC-MRD at TP2 in a separate analysis of LR (**d**), IR (**e**) and HR (**f**) patients. EFS, event-free survival; MFC, multiparametric flow cytometry; MRD, minimal residual disease; TP1, after the first induction course; TP2, at the end of the second induction course (before start of consolidation)

When using a cutoff level of 0.1%, the MFC-MRD level at TP1 was not prognostically significant for EFS in a separate analysis of LR, IR, and HR patients (Fig. 5a–c). Additionally, in a separate analysis of LR patients, the MFC-MRD level at TP2 was not significantly different (100.0% ± 0.0% versus 88.6% ± 3.7%, P = 0.440) (Fig. 5d). However, 5-year EFS of HR and IR patients in the MFC-MRD ≥ 0.1% subgroup at TP2 was significantly lower than that in the MFC-MRD < 0.1% subgroup (IR: 56.2% ± 9.3% *vs*. 76.1% ± 3.0%, *P* = 0.012; HR: 33.2% ± 9.0% *vs*. 53.6% ± 6.7%, *P* = 0.002) (Fig. 5e, f).

Additionally, we explored the prognostic significance of MFC-MRD levels at TP2 in different genetic subtypes. MFC-MRD levels at TP2 predicted the prognosis of patients with *KMT2A* rearrangement: the MFC-MRD ≥ 0.1% subgroup had an EFS of 44.0% ± 14.2% at 5 years, whereas the MFC-MRD < 0.1% subgroup had an EFS of 75.4% ± 5.6% (*P* < 0.001) (Supplementary Fig. 5a, Additional file). With other common genetic abnormalities, i.e., the presence of *RUNX1-RUNX1T1*, *FLT3-ITD* mutation, and *ASXL1* mutation, MFC-MRD levels at TP2 were not significantly related to the final outcomes (Supplementary Fig. 5b–d, Additional file).

### *Impact of HSCT during the first complete remission (CR1) in patients with MFC-MRD* ≥ *0.1% at TP2*

**Fig. 6:**
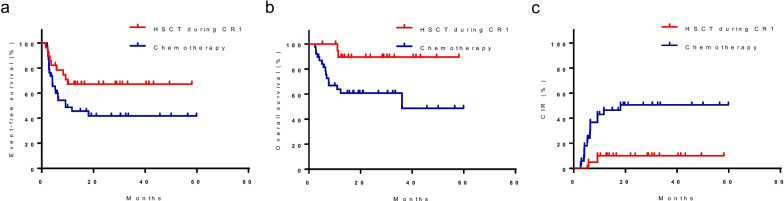
Survival probability by different treatments after CR1 in patients with MRD < 0.1% before consolidation. EFS (**a**), OS (**b**), and CIR (**c**) of the patients who underwent HSCT during CR1 and those who only received consolidation chemotherapy. CR1, the first complete remission; EFS, event-free survival; OS, overall survival; CIR, cumulative incidence of relapse; MFC, multiparametric flow cytometry; MRD, minimal residual disease

Of the 66 patients in the MFC-MRD ≥ 0.1% subgroup at the end of the second induction course (before consolidation), 7 received salvage transplantation, 21 underwent HSCT during CR1, and 38 received consolidation chemotherapy only. Patients in the MFC-MRD ≥ 0.1% subgroup who underwent HSCT during CR1 had a significantly higher 5-year EFS and OS and lower CIR than those who only received consolidative chemotherapy (EFS: 90.0% ± 6.7% *vs*. 41.8% ± 8.4%, *P* < 0.001; OS: 89.5% ± 7.0% *vs.* 60.7% ± 8.2%, *P* = 0.011; CIR: 10.0% ± 6.7% *vs*. 50.5% ± 9.1%, *P* = 0.004) (Fig. 6a–c).

## Discussion

Currently, the predominant treatment for pediatric AML involves a multidrug induction regimen based on cytarabine and anthracycline, followed by post-remission consolidative chemotherapy or HSCT [1, 20–23]. Apart from considering cytogenetic and molecular aberrations, the response to induction chemotherapy was an important factor in determining the intensity of subsequent therapy and selecting candidates for HSCT [7, 20–22]. Currently, an important question in pediatric AML revolves around the choice of method for evaluating therapeutic response.

Owing to the low sensitivity and specificity of morphological assessment and the limited applicability of RT-PCR of fusion transcripts in pediatric patients with AML (applicable in only 50%–60% of cases), MFC-MRD is now a generally accepted approach in evaluating treatment response [6–12, 19, 24–28]. With the largest cohort of pediatric patients with AML treated according to a uniform protocol in China, the current study demonstrates the prognostic significance of MFC-MRD after induction chemotherapy. Our study revealed that MFC-MRD following induction therapy, particularly after the second session of induction, has the potential to serve as a prognostic indicator for children with AML. Additionally, this measurement can aid in the stratification of post-remission treatment strategies.

Numerous studies have confirmed the importance of MFC-MRD for relapse risk and subsequent prognosis [4, 7, 9, 13–15, 26–29]. However, further investigation is needed to determine the ideal MFC-MRD time points and cutoff values for distinguishing patients with different prognoses. The AML02 multicenter trial and Children’s Oncology Group study showed that using a cutoff level of 1.0%, MFC-MRD at the end of the first induction course could predict the final outcomes [8, 9]. In a prospective Children’s Cancer Group study and a single trial (United Kingdom Medical Research Council AML12 and similar Dutch Childhood Oncology Group ANLL97), MFC-MRD ≥ 0.5% after the first course of chemotherapy predicted a poor outcome [26, 29]. In an international prospective study, MFC-MRD, either ≥ 1% or ≥ 0.1% at early time points of follow-up (until day 84), especially on day 28 after diagnosis, could be a significant predictor of 3-year EFS [14]. Although using the same cutoff point of 0.1%, the study of Associazione Italiana di Ematologia e Oncologia Pediatrica (AIEOP)-AML 2002/01 showed that MFC-MRD ≥ 0.1% after the first induction course was an independent adverse prognostic factor for disease-free survival [13], while the result of Nordic Society of Paediatric Haemato-Oncology (NOPHO) AML 2004 study showed that MFC-MRD ≥ 0.1% after the second course induction (before consolidation therapy) was an independent adverse prognostic factor for EFS and OS [15].As a cutoff point of 0.1% has been included and found to be relevant in most published studies to date, this cutoff point is most commonly recommended to define positive MFC-MRD [10, 18, 25, 30].

Our data revealed that patients with a MFC-MRD level of 0.1% or above at TP2 had a recurrence incidence of up to 40%. Furthermore, the results of amultivariate analysis indicated that the MFC-MRD level at the end of the second induction course was an independent risk factor. Based on prior publications and our own research, it is reasonable to consider the 0.1% threshold value as appropriate. Furthermore, our data indicate that the prognostic significance of MFC-MRD at TP2 surpasses that of measuring it at TP1.

To date, genetic/molecular characteristics have been the most important basis for conventional risk group stratification [3, 20]. Currently, the number of HR markers has markedly increased compared with the number of good-risk markers [31]. Nonetheless, even with the same fusion gene or gene mutation, the prognosis of patients in the same risk group may be quite distinct [32]. In our study, MFC-MRD measurements at the end of the second induction course were widely applicable and could further differentiate the prognosis of IR and HR patients, especially patients with *KMT2A*-rearrangement, which is consistent with the conclusion of the latest research the International Berlin-Frankfurt-Münster Study Group [33]. However, these measurements could not further predict the prognosis of patients with other common genetic abnormality, i.e., LR, *RUNX1-RUNX1T1*, *FLT3-ITD* mutation, and *ASXL1* mutation. These results may be attributable to a small sample of a particular subtype or to variations in treatments.

The incorporation of MFC-MRD in clinical practice has the potential to offer valuable prognostic information and enhance existing pretreatment factors such as cytogenetics and genomic alterations. However, there exists a debate regarding the routine use of MFC-MRD analysis, specifically about the utilization of HSCT or hypomethylating agents for certain patient subgroups who are in morphological remission but exhibit MFC-MRD positivity [34]. In the multicenter AML 02 trial, therapy after the first induction course was directed based on the assessment of d22 MFC-MRD, and patients with AML achieved a 3-year EFS of 63% and an OS of 71%, representing substantial gains over the results of trials conducted in the USA [7]. In the ongoing AIEOP AML 2013 study [13] and AIEOP-BFM AML 2020 study [1], MFC-MRD has been used to guide the intensification of treatment. Regardless of genetics, patients with MFC-MRD ≥ 1% at the end of the first induction course or MFC-MRD ≥ 0.1% at the end of the second induction course should be stratified into the HR group, and patients with MFC-MRD ≥ 0.1% and < 1% at the end of the first induction course and MFC-MRD < 0.1% at the end of the second induction should be stratified into an IR group. However, the results of the AIEOP AML 2013 study have not been reported yet. The outcomes of patients positive for MFC-MRD are relatively poor, regardless of whether HSCT is performed or not; however it can improve with HSCT [35–38]. Our results also showed that the OS and EFS of patients with MFC-MRD ≥ 0.1% after the second induction course who received HSCT during CR1 were significantly higher than those of patients who only received consolidative chemotherapy. We demonstrated that HSCT during CR1 may improve the long-term outcome of patients with detectable RD after induction. However, establishing the cutoff level and assessment time points of MFC-MRD to guide post-remission therapy requires more prospective studies.

This study has certain limitations, including its retrospective nature, certain therapy delays, and some missing MFC-MRD data. Although we offered standardized training for data gathering, further enhancements in data collection and quality control are necessary for further improved in the future improvements. In the future, a prospective multicenter clinical trial, guided by the insights from this study and characterized by rigorous quality control measures, will be necessary to establish the clinical prognostic utility and reliability of MFC-MRD.

## Conclusions

The findings of our study provide confirmation that the MFC-MRD level at TP2 is strongly correlated with unfavorable outcomes in children with AML. With its enhanced precision and sensitivity compared to morphological evaluation, MFC-MRD can serve as a valuable supplement to genetic abnormalities in directing post-remission therapies. Nevertheless, the therapeutic implications of MFC-MRD monitoring remain incompletely understood. Therefore, it is imperative that randomized studies prioritize investigating whether the incorporation of MFC-MRD monitoring into clinical practice provides advantages in the treatment of AML.

### Supplementary Information

**Additional file 1:** **Supplementary Figure 1.** Treatment schema for the C-HUANAN-AML 15 protocol. *Note: intermediate-risk patients with a sibling donor and high-risk patients were advised to undergo allo-HSCT; Children aged <1 year had all chemotherapy doses reduced by 25%. HSCT, hematopoietic stem cell transplantation. **Supplementary Figure 2.** EFS by MFC-MRD status after the first course of induction and before the start of consolidation. According to MFC-MRD levels, patients were stratified into three MFC-MRD-based groups (< 0.01%, 0.01%–0.1%, and ≥ 0.1%). (a) EFS by MFC-MRD status after the first course of induction. (b) EFS by MFC-MRD status before the start of consolidation. EFS, event-free survival; MFC, multiparametric flow cytometry; MRD, minimal residual disease. **Supplementary Figure 3.** Survival probability by morphological response after the first course of induction and before the start of consolidation. According to the morphological response, patients were stratified into two morphology-based groups (CR; non-CR). EFS (a), OS (b), and CIR (c) according to morphological response after the first induction course; EFS (d), OS (e), and CIR (f) according to morphological response before start of consolidation. EFS, event-free survival; OS, overall survival; CIR, cumulative incidence of relapse; MFC, multiparametric flow cytometry; MRD, minimal residual disease. **Supplementary Figure 4.** Survival probability by MFC-MRD status in a separate analysis of patients with ≥5% or <5% blasts, based on morphology after the first induction course. According to MFC-MRD levels, patients were stratified into two MFC-MRD-based groups (MRD < 0.1%; MRD ≥ 0.1%). EFS (a), OS (b), and CIR (c) according to MFC-MRD level in a separate analysis of patients with <5% blasts based on morphology after the first induction course (EFS: 38.5 ± 13.5% *versus *45.0 ± 6.9%, *P* = 0.603; OS: 50.5 ± 7.5% *versus *75.2 ± 12.6%, *P* = 0.280; CIR: 34.7 ± 7.9% *versus *29.3 ± 14.3%, *P* = 0.704). EFS (d), OS (e), and CIR (f) according to MFC-MRD level in a separate analysis of patients with ≥5% blasts based on morphology after the first induction course (EFS: 70.0 ± 4.4% *versus *72.8 ± 2.7%, *P* = 0.744; OS: 75.8 ± 4.2% *versus *80.1 ± 2.5%, *P* = 0.474; CIR: 25.5 ± 4.4% *versus *20.6 ± 2.6%, *P* = 0.388). EFS, event-free survival; OS, overall survival; CIR, cumulative incidence of relapse; MFC, multiparametric flow cytometry; MRD, minimal residual disease. **Supplementary Figure 5.** Survival probability by MFC-MRD status before the start of consolidation in a separate analysis of patients with different common genetic abnormalities. According to MFC-MRD levels, patients were stratified into two MFC-MRD-based groups (MRD < 0.1%; MRD ≥ 0.1%). EFS according to MFC-MRD before starting consolidation in a separate analysis of patients with *KMT2A*-rearrangement (a), *RUNX1-RUNX1T1 *(b), *FLT3-ITD* mutation (c), and *ASXL1* mutation (d). EFS, event-free survival; MFC, multiparametric flow cytometry; MRD, minimal residual disease. **Supplementary Table 1.** Number of patients recruited at each center. **Supplementary Table 2.** Characteristics of the patients. **Supplementary Table 3.** Prognostic risk systems in the C-HUANAN-AML15 protocol. **Supplementary Table 4.** Antibody panel used for immunophenotype studies at diagnosis. **Supplementary Table 5.** List of hospitals for MFC-MRD detection at the two MFC core hubs. **Supplementary Table 6.** Most common five-color combinations used to assess MRD after the first and second induction courses (chnam MRD panel).

## Data Availability

The datasets supporting the conclusions of this article are not publicly available due to data protection for enrolled centers but are available from the corresponding author on reasonable request.

## Abbreviations

AIEOP: Associazione Italiana di Emato Oncologia Pediatrica
AML: Acute myeloid leukemia
CBF: Core-binding factor
CIR: Cumulative incidence of relapse
CNS: Central nervous system
CR: Complete remission
DFS: Disease-free survival
EFS: Event-free survival
ELN: European LeukemiaNet
HR: High-risk
HSCT: Hematopoietic stem cell transplantation
IR: Intermediate-risk
LR: Low-risk
MFC: Multiparametric flow cytometry
MRD: Minimal residual disease
OS: Overall survival
