# Supplementary material for: Prognostic significance of multiparametric flow cytometry minimal residual disease at two time points after induction in pediatric acute myeloid leukemia
Source: BMC Cancer. 2024 Jan 9;24:46. doi: 10.1186/s12885-023-11784-4 (PMC10775489; doi:10.1186/s12885-023-11784-4)
Supplement: Supplementary file 1 — Additional file 1: Supplementary Figure 1. Treatment schema for the C-HUANAN-AML 15 protocol. *Note: intermediate-risk patients with a sibling donor and high-risk patients were advised to undergo allo-HSCT; Children aged <1 year had all chemotherapy doses reduced by 25%. HSCT, hematopoietic stem cell transplantation. Supplementary Figure 2. EFS by MFC-MRD status after the first course of induction and before the start of consolidation. According to MFC-MRD levels, patients were stratified into three MFC-MRD-based groups (< 0.01%, 0.01%–0.1%, and ≥ 0.1%). (a) EFS by MFC-MRD status after the first course of induction. (b) EFS by MFC-MRD status before the start of consolidation. EFS, event-free survival; MFC, multiparametric flow cytometry; MRD, minimal residual disease. Supplementary Figure 3. Survival probability by morphological response after the first course of induction and before the start of consolidation. According to the morphological response, patients were stratified into two morphology-based groups (CR; non-CR). EFS (a), OS (b), and CIR (c) according to morphological response after the first induction course; EFS (d), OS (e), and CIR (f) according to morphological response before start of consolidation. EFS, event-free survival; OS, overall survival; CIR, cumulative incidence of relapse; MFC, multiparametric flow cytometry; MRD, minimal residual disease. Supplementary Figure 4. Survival probability by MFC-MRD status in a separate analysis of patients with ≥5% or <5% blasts, based on morphology after the first induction course. According to MFC-MRD levels, patients were stratified into two MFC-MRD-based groups (MRD < 0.1%; MRD ≥ 0.1%). EFS (a), OS (b), and CIR (c) according to MFC-MRD level in a separate analysis of patients with <5% blasts based on morphology after the first induction course (EFS: 38.5 ± 13.5% versus 45.0 ± 6.9%, P = 0.603; OS: 50.5 ± 7.5% versus 75.2 ± 12.6%, P = 0.280; CIR: 34.7 ± 7.9% versus 29.3 ± 14.3%, P = 0.704). EFS (d), OS (e), an [file 12885_2023_11784_MOESM1_ESM.docx]

**Additional file**


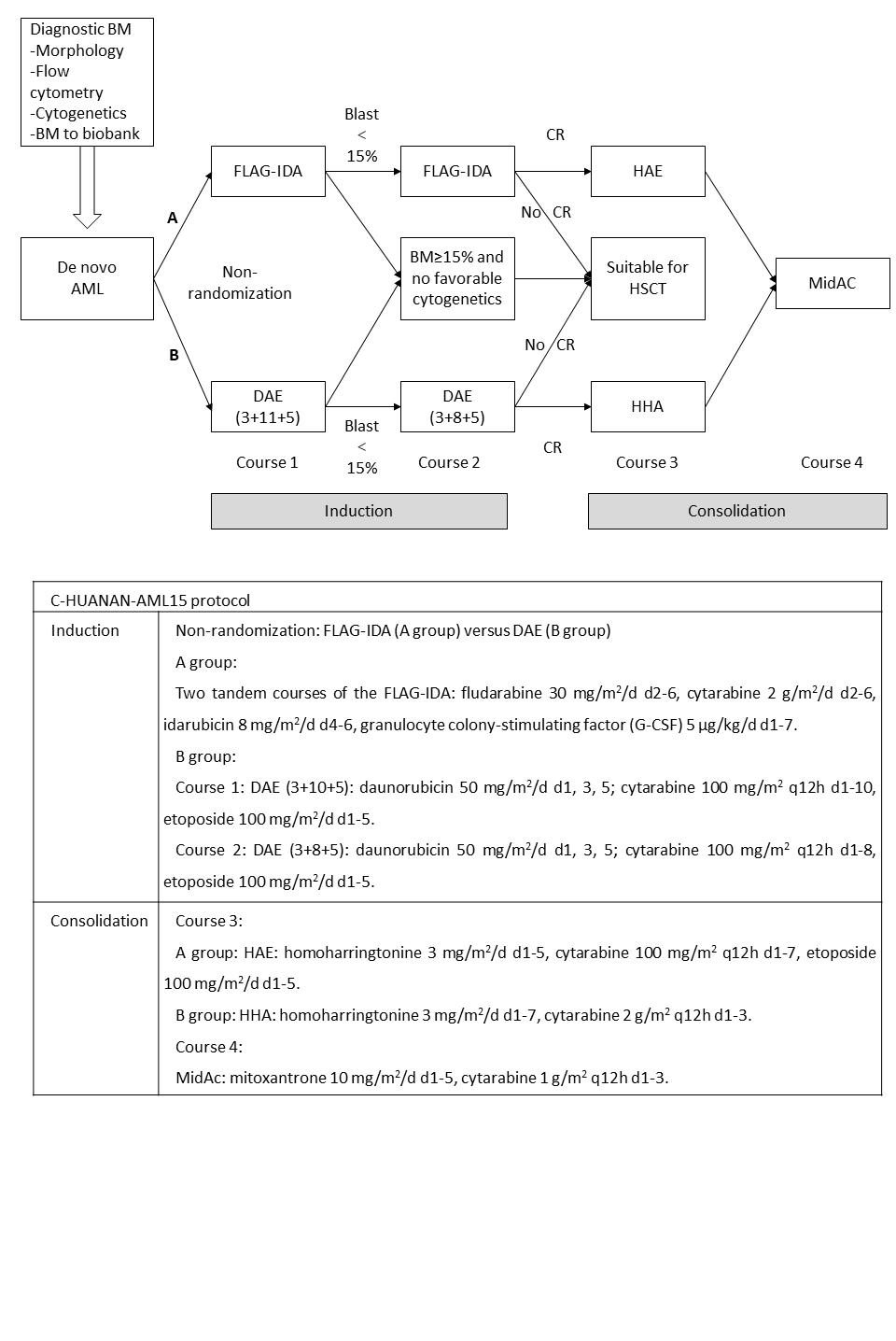


Supplementary Figure 1. Treatment schema for the C-HUANAN-AML 15 protocol. *Note: intermediate-risk patients with a sibling donor and high-risk patients were advised to undergo allo-HSCT; Children aged <1 year had all chemotherapy doses reduced by 25%. HSCT, hematopoietic stem cell transplantation.


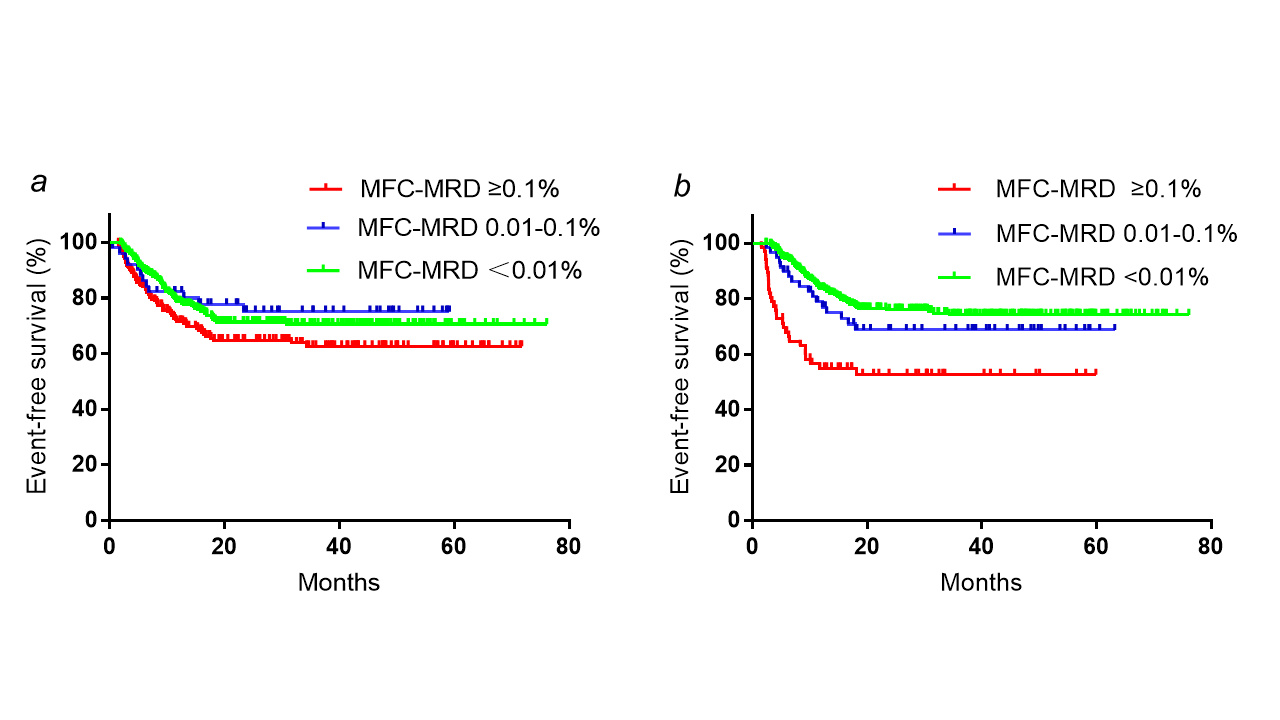


Supplementary Figure 2. EFS by MFC-MRD status after the first course of induction and before the start of consolidation. According to MFC-MRD levels, patients were stratified into three MFC-MRD-based groups (< 0.01%, 0.01%–0.1%, and ≥ 0.1%). (a) EFS by MFC-MRD status after the first course of induction. (b) EFS by MFC-MRD status before the start of consolidation. EFS, event-free survival; MFC, multiparametric flow cytometry; MRD, minimal residual disease


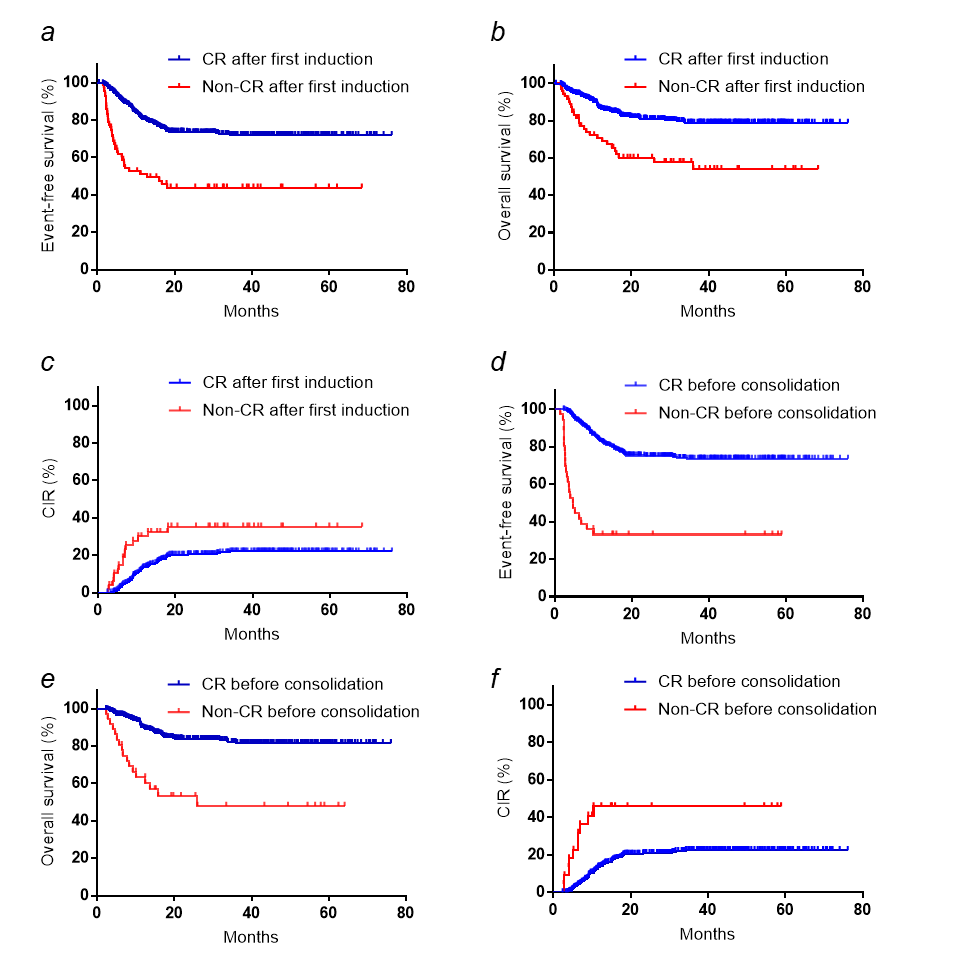
 Supplementary Figure 3. Survival probability by morphological response after the first course of induction and before the start of consolidation. According to the morphological response, patients were stratified into two morphology-based groups (CR; non-CR). EFS (a), OS (b), and CIR (c) according to morphological response after the first induction course; EFS (d), OS (e), and CIR (f) according to morphological response before start of consolidation. EFS, event-free survival; OS, overall survival; CIR, cumulative incidence of relapse; MFC, multiparametric flow cytometry; MRD, minimal residual disease.


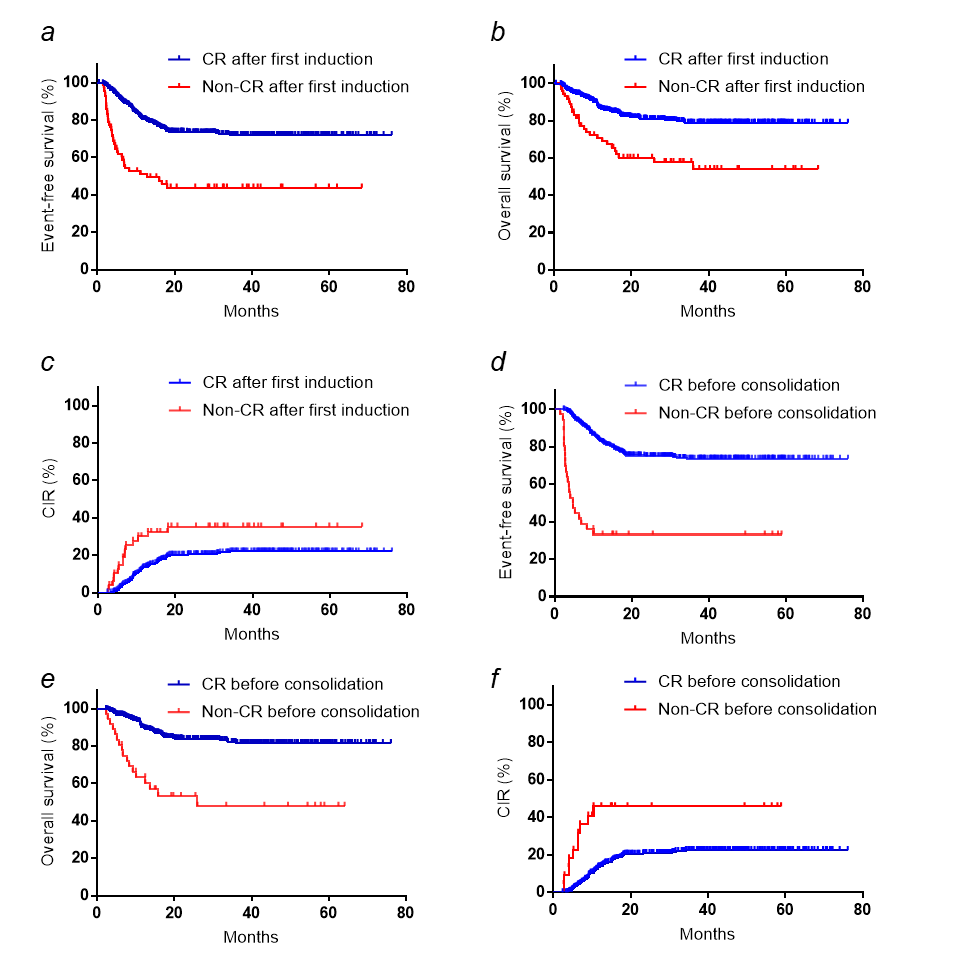


Supplementary Figure 4. Survival probability by MFC-MRD status in a separate analysis of patients with ≥5% or <5% blasts, based on morphology after the first induction course. According to MFC-MRD levels, patients were stratified into two MFC-MRD-based groups (MRD < 0.1%; MRD ≥ 0.1%). EFS (a), OS (b), and CIR (c) according to MFC-MRD level in a separate analysis of patients with <5% blasts based on morphology after the first induction course (EFS: 38.5 ± 13.5% *versus* 45.0 ± 6.9%, *P* = 0.603; OS: 50.5 ± 7.5% *versus* 75.2 ± 12.6%, *P* = 0.280; CIR: 34.7 ± 7.9% *versus* 29.3 ± 14.3%, *P* = 0.704). EFS (d), OS (e), and CIR (f) according to MFC-MRD level in a separate analysis of patients with ≥5% blasts based on morphology after the first induction course (EFS: 70.0 ± 4.4% *versus* 72.8 ± 2.7%, *P* = 0.744; OS: 75.8 ± 4.2% *versus* 80.1 ± 2.5%, *P* = 0.474; CIR: 25.5 ± 4.4% *versus* 20.6 ± 2.6%, *P* = 0.388). EFS, event-free survival; OS, overall survival; CIR, cumulative incidence of relapse; MFC, multiparametric flow cytometry; MRD, minimal residual disease.


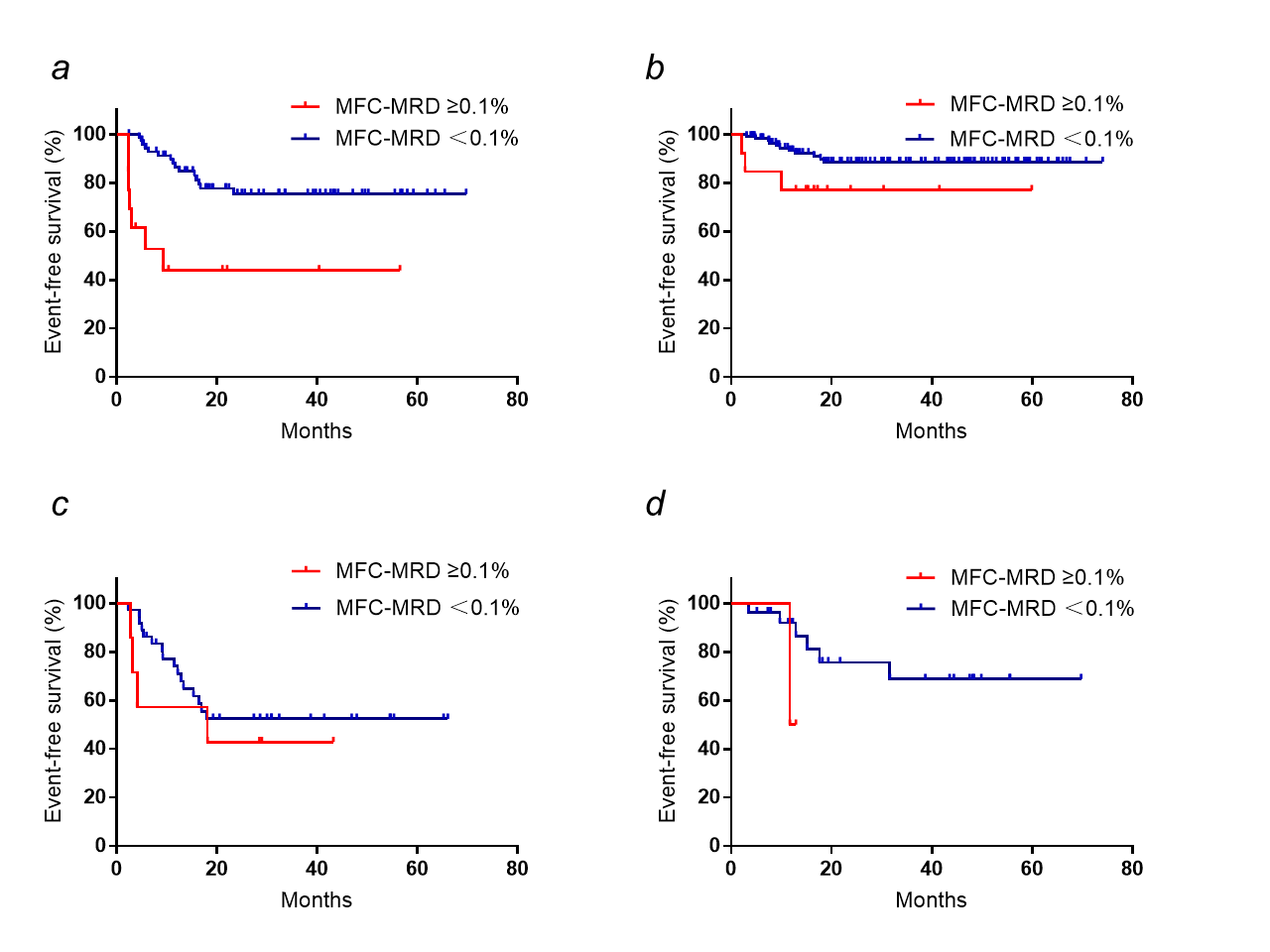
 Supplementary Figure 5. Survival probability by MFC-MRD status before the start of consolidation in a separate analysis of patients with different common genetic abnormalities. According to MFC-MRD levels, patients were stratified into two MFC-MRD-based groups (MRD < 0.1%; MRD ≥ 0.1%). EFS according to MFC-MRD before starting consolidation in a separate analysis of patients with *KMT2A*-rearrangement (a), *RUNX1-RUNX1T1* (b), *FLT3-ITD* mutation (c), and *ASXL1* mutation (d). EFS, event-free survival; MFC, multiparametric flow cytometry; MRD, minimal residual disease.

Supplementary Table 1. Number of patients recruited at each center.

| Hospital | Number (%) |
| --- | --- |
| Fujian Medical University Union Hospital | 164 (28.1) |
| Southern Medical University Nanfang Hospital | 95 (16.3) |
| Hunan Children's Hospital | 80 (13.7) |
| Shenzhen Children’s Hospital | 60 (10.3) |
| Zhujiang Hospital of Southern Medical University | 53 (9.1) |
| Guangzhou Women and Children’s Medical Center | 49 (8.4) |
| People's Hospital of Hunan Province | 35 (6.0) |
| Sun Yat-sen Memorial Hospital | 27 (4.6) |
| The First Affiliated Hospital of Xiamen University | 21 (3.6) |
| Total | 584 (100.0) |

Supplementary Table 2. Characteristics of the patients.

| Number of patients enrolled onto the C-HUANAN-AML 15 study | 584 |
| --- | --- |
| Sex, n (%) |  |
| Male | 326 (55.8) |
| Female | 238 (44.2) |
| Age at diagnosis |  |
| Median | 71 months |
| Range | 2–176 months |
| n (%) |  |
| <12 months | 26 (4.5) |
| 12–24 months | 79 (13.5) |
| 24–120 months | 358 (61.3) |
| 120–168 months | 121 (20.7) |
| WBC at diagnosis (× 10^9^/L) |  |
| Median | 22.8 |
| Range | 0.1–464.2 |
| n (%) |  |
| ≥50 × 10^9^/L | 179 (30.7) |
| <50 × 10^9^/L | 405 (69.3) |
| FAB classification, n (%) |  |
| M0 | 13 (2.2) |
| M1 | 15 (2.6) |
| M2 | 194 (33.2) |
| M4 | 39 (6.7) |
| M5 | 200 (34.2) |
| M6 | 4 (0.7) |
| M7 | 40 (6.8) |
| Unknown | 75 (12.8) |
| *RUNX1-RUNX1T1* | 155 (26.5) |
| *CBFB-MYH11* | 39 (6.7) |
| *KMT2A* rearranged | 108 (18.5) |
| *MLLT3-KMT2A* | 46 (7.9) |
| Complex karyotype | 26 (4.5) |
| -7 or 7q- | 21 (3.6) |
| *C-KIT* mutation | 64 (11.0) |
| *FLT3-ITD* mutation | 59 (10.1) |
| *ASXL1* mutation | 36 (6.2) |
| *NPM1* mutation | 12 (2.1) |
| Biallelic mutated *CEBPA* mutation | 18 (3.1) |
| Risk stratification according to |  |
| 2017 ELN criteria, n (%) |  |
| HR | 215 (36.8) |
| IR | 192 (32.9) |
| LR | 177 (30.3) |

AML, acute myeloid leukemia; WBC, white blood cell; FAB, French-American-British; CR, complete remission; ELN, European LeukemiaNet; HR, high risk; IR, intermedium risk; LR, low risk; ELN, European LeukemiaNet.

Supplementary Table 3. Prognostic risk systems in the C-HUANAN-AML15 protocol.

| Risk stratification | Genetic abnormality and induction chemotherapy response |
| --- | --- |
| Low risk | Include one of the following genetic abnormality and CR after the first induction course:  t(8;21)(q22;q22); AML/ETO (RUNX1- RUNX1T1); inv(16)(p13q22)/t(16;16)(p13;q22); CBFB-MYH11; normal cytogenetics: NPM1 or isolated biallelic (double) CEBPA mutation in the absence of FLT3-ITD |
| Intermediate risk | Exclude low risk or high risk genetic abnormality and blast in bone < 15% after the first induction course and CR after the second induction course |
| High risk | Include one of the following genetic abnormal blast in bone ≥ 15% after the first induction course or no CR after the second induction course:  Mutated FLT3-ITD;  Complex karyotype;  -5 or del(5q);  abn(3q);  abn(17p);  -7 or del(7q) |

CR, complete remission.

Supplementary Table 4. Antibody panel used for immunophenotype studies at diagnosis.

|  | *FITC* | *PE* | *PE-Cy5.5* | *PE-Cy7* | *APC* | *APC-A750* | *V510* | *PB* |
| --- | --- | --- | --- | --- | --- | --- | --- | --- |
| 1 | CD5 | CD7 | CD56 | CD8 | CD4 | CD3 | CD45 | CD2 |
| 2 | KAPPA | LAMBDA | CD5 | CD10 | CD19 |  | CD45 | CD20 |
| 3 | CD14 | CD13 | CD64 | CD16 | CD11b |  | CD45 | CD15 |
| 4 | CD36 | CD33 | CD34 | CD117 | CD71 |  | CD45 | HLA-DR |
| 5 | CD38 | CD117 | CD56 | CD19 | CD138 |  | CD45 | CD200 |
| 6 | CD61 |  | 7AAD |  |  |  | CD45 |  |
| 7 | cMPO | cμ or cCD79a | CD34 | CD117 | cCD22 | CD19 | CD45 | cCD3 |
| 8 | cIgG1 | cIgG1 | CD34 | CD117 | cIgG1 | CD19 | CD45 | cIgG1 |

Supplementary Table 5. List of hospitals for MFC-MRD detection at the two MFC

core hubs.

| MFC core hub | Kindstar Globalgene Technology, Inc, China | KingMed Diagnostics Group Co., Ltd, China |
| --- | --- | --- |
| Hospital list | 1. Fujian Medical University Union Hospital  2. Hunan Children’s Hospital  3. Guangzhou Women and Children’s Medical Center  4. Hunan Provincial People’s Hospital | 1. Nanfang Hospital, Southern Medical University  2. Shenzhen Children’s Hospital  3. Zhujiang Hospital of Southern Medical University  4. Sun Yat-sen Memorial Hospital  5. The First Affiliated Hospital of Xiamen University |

MFC-MRD, multiparametric flow cytometry-minimal residual disease.

Supplementary Table 6. Most common five-color combinations used to assess MRD after the first and second induction courses (chnam MRD panel).

|  | FITC | PE | ECD | PE-Cy5 | PE-Cy7 |
| --- | --- | --- | --- | --- | --- |
| 1 | IgG1 | IgG1 | IgG1 | IgG1 | CD45 |
| 2 | HLA-DR | CD33 | CD34 | CD117 | CD45 |
| 3 | CD11b | CD13 or CD7 | CD34 | CD15 | CD45 |
| 4 | CD10 | CD56 | CD34 | CD19 | CD45 |
| 4. AML-M4/M5 | CD36 | CD64 | CD14 | CD56 | CD45 |
| 5 | CD71 |  | CD41 | 7AAD | CD45 |

MRD, minimal residual disease; AML, acute myeloid leukemia; PE, P-phycoerythrin; FITC, Fluorescein Isothiocyanate; ECD, PE-TR(PE-TexasRed)/ECDPE-TR.
